# Carbonic anhydrase under pressure

**DOI:** 10.1107/S2052252517018012

**Published:** 2018-01-01

**Authors:** Anders Liljas

**Affiliations:** a Department of Biochemistry and Structural Biology, Lund University, Lund, Sweden

**Keywords:** carbonic anhydrase II, proton transfer, water dynamics, high pressure

## Abstract

Investigations of the rapid enzyme carbonic anhydrase have now been extended by crystallographic analysis at high CO_2_ pressures to examine the movements of water molecules in different steps of the catalysis. The rate of catalysis seems well explained by the assembled observations.

Carbonic anhydrase is among the most thoroughly investigated enzymes. It catalyzes the conversion between carbon dioxide and bicarbonate 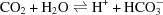



Early on it was realised that the rate of the spontaneous reaction was far too low to function physiologically. Thus, there must be an enzyme and it was identified by Meldrum & Roughton (1932 ▸). Now six distinct families of the enzyme are known (α, β, γ, δ, ζ and η). Furthermore, the human α-form is divided into 14 different isoenzymes with the same basic fold but with different cellular locations and properties. Human CA II (hCAII) is expressed in many cell types and is involved in many physiological processes. Its catalytic rate is limited by the rate of diffusion of substrates and products (Frost & McKenna, 2013 ▸). Evidently, the substrates or the products cannot bind strongly and the conformational changes need to be minimal.

The first structure of hCAII provided the protein fold and the active-site zinc ion in a 15 Å deep cleft. A zinc-bound water (W_Zn_) and a number of additional active-site water molecules were identified (Liljas *et al.*, 1972 ▸). Numerous studies of mutants and bound inhibitors have provided further insights (Liljas *et al.*, 1994 ▸). In the enzyme catalysis the W_Zn_ is the hydration water. It is activated by releasing a proton through the water structure to a histidine in the active site, His64, from where it is released into bulk solvent (Nair & Christianson, 1991 ▸). The p*K_a_* of W_Zn_ is around 7, as it is for His64 (Domsic *et al.*, 2010 ▸). W_Zn_ is hydrogen bonded to Oγ1 of Thr199, which is further hydrogen bonded to the negatively charged Glu106. This forces the zinc ligand to provide a proton for the hydrogen bond to Thr199. The CO_2_ is bound in the deep hydro­phobic part of the active site next to W_Zn_ (Kim *et al.*, 2005 ▸; Domsic *et al.*, 2008 ▸). The hydration of CO_2_ or dehydration of HCO_3_^−^ is efficiently done without significant structural changes to the protein. The entry or dissipation of CO_2_ probably occurs along the hydro­phobic side of the active site while HCO_3_^−^ enters or leaves along the hydro­philic side. However, the activities in the active site during catalysis primarily need the dynamics of water molecules.

An article in the current issue of **IUCrJ** describes crystallographic analyses of hCAII at ultrahigh resolution where the crystals were exposed to CO_2_ under 7 and 2.5 atm pressure (Kim *et al.*, 2018 ▸). These studies extend previous studies where the crystals were exposed to 15 atm pressure of CO_2_ and frozen to liquid nitro­gen temperature after various lengths of time (Kim *et al.*, 2016 ▸). The previous investigations provided the location of a fully bound CO_2_ molecule at the expected position at the bottom of the active site. The present article illuminates the previous courageous high-pressure experiments with studies at intermediate pressure. The observations lead to a range of intermediate structures with lower occupancy of substrate and identify what rearrangements take place at partial occupancy of substrate. A unique feature of the article is the focus on rearrangements of the water structure during catalysis. This is something of importance for most enzymes.

An obvious rearrangement is the replacement of CO_2_ by two water molecules, called ‘deep water’ (W_DW_ and W_DW_′). They substitute for the two O atoms of CO_2_. The flexibility of the network of water molecules in the active site is described in detail. The waters adjust in several steps to the changes that occur in the active site. Several water molecules also appear to have neighbouring alternate positions.

The most dramatic conformational change is the flipping of His64 from an inward-facing orientation to an orientation towards bulk solvent to release a proton from the zinc water. Having received the proton, the positively charged imidazole is probably repelled by the positive charge of the zinc ion to adopt the outward conformation to release the proton to bulk water. Subsequently it flips back to the original position.

The distance from W_Zn_ to His64 is too long for a direct proton transfer. Rather, the proton is shuttled from W_Zn_ to His64 *via* two bridging water molecules (W1 and W2; Fig. 1 ▸ *a*). The series of intermediate structures studied show that the water molecule next to W_Zn_ (W1) is absent when His64 is in the outward position (Fig. 1 ▸ *b*). In this state there can be no proton transfer, since His 64 is already protonated. Instead of W1 an intermediate water molecule, W_I_, appears. It also has an alternate site W_I_′.

 A new cycle of catalysis begins with a new W_Zn_ that replaces the bicarbonate. The site can be filled from any of several neighbour water molecules. W_Zn_ then gets deprotonated and CO_2_ binds by displacing the two deep-water molecules (W_DW_ and W_DW_′). W1 and W2 then occupy their normal positions where W2, closest to His64, has two different positions, W2 and W2′. The second position, W2′, is related to the flipping of the histidine to the outward conformation, which allows more space.

His64 then accepts the proton from W_Zn_ through W1 and W2. At this stage His64 adopts its outward facing conformation to release its proton, which leads to changes in the water structure. W_Zn_ is now able to make a nucleophilic attack on the carbon of the carbon dioxide to yield bicarbonate (Fig. 1 ▸ *c*). The binding of the bicarbonate is not optimal since it is liganded to the zinc ion by its protonated oxygen but not a negatively charged one. The protonated oxygen is forced to remain at the zinc due to its hydrogen bond to the obligate hydrogen-bond acceptor Oγ1 of Thr199. This makes the binding weak, and three water molecules then readily displace the bicarbonate. This completes the catalytic process.

The high rate of the enzyme is evidently due to the weak binding of the substrates, as well as the firm structure of its active site, the only moving part being His64. Easily moving water molecules during catalysis are also a requirement for the high rate.

## Figures and Tables

**Figure 1:**
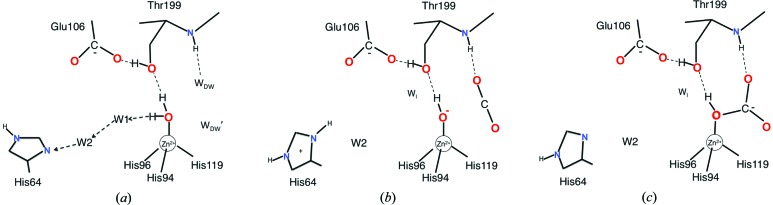
A likely catalytic mechanism for human carbonic anhydrase II. In the first step (*a*) the zinc water (W_Zn_) releases a proton to His64 *via* W1 and W2. When carbon dioxide binds (*b*) it releases the two deep-water molecules. W1 disappears and is replaced by W_I_. His64 adopts the outward orientation to release the abstracted proton to bulk solvent. Then the W_Zn_ makes a nucleophilic attack on the carbon of the carbon dioxide to form bicarbonate (*c*). Three water molecules rapidly replace the bicarbonate and His64 returns to its inward orientation (*a*). Several additional water molecules are identified and participate in the dynamic water structure of the active site.

## References

[bb1] Domsic, J. F., Avvaru, B. S., Kim, C. U., Gruner, S. M., Agbandje-McKenna, M., Silverman, D. N. & McKenna, R. (2008). *J. Biol. Chem.* **283**, 30766–30771. 10.1074/jbc.M805353200 PMC2576554 18768466

[bb2] Domsic, J. F., Williams, W., Fisher, S. Z., Tu, C., Agbandje-McKenna, M., Silverman, D. N. & McKenna, R. (2010). *Biochemistry*, **49**, 6394–6399. 10.1021/bi1007645 PMC2925223 20578724

[bb3] Frost, S. C. & McKenna, R. (2013). *Carbonic anhydrase: mechanism, regulation, links to disease, and industrial applications.* Dordrecht: Springer Science and Business Media.

[bb4] Kim, C. U., Kapfer, R. & Gruner, S. M. (2005). *Acta Cryst.* D**61**, 881–890. 10.1107/S090744490500836X 15983410

[bb10] Kim, J. K., Lomelino, C. L., Avvaru, B. S., Mahon, B. P., McKenna, R., Park, S. & Kim, C. U. (2018). *IUCrJ*, **5**, 93–102. 10.1107/S2052252517017626 PMC5755581 29354275

[bb5] Kim, C. U., Song, H., Avvaru, B. S., Gruner, S. M., Park, S. & McKenna, R. (2016). *Proc. Natl. Acad. Sci. USA*, **113**: 5257–5262. 10.1073/pnas.1520786113 PMC4868437 27114542

[bb6] Liljas, A., Håkansson, K., Jonsson, B. H. & Xue, Y. (1994). *Eur. J. Biochem.* **219**, 1–10. 10.1007/978-3-642-79502-2_1 8306976

[bb7] Liljas, A., Kannan, K. K., Bergstén, P.-C., Waara, I., Fridborg, K., Strandberg, B., Carlbom, U., Järup, L., Lövgren, S. & Petef, M. (1972). *Nature New Biol.* **235**, 131–137. 10.1038/newbio235131a0 4621826

[bb8] Meldrum, N. U. & Roughton, F. J. W. (1932). *J. Phys.* **80**, 113–142. 10.1113/jphysiol.1933.sp003077 PMC1394121 16994489

[bb9] Nair, S. K. & Christianson, D. W. (1991). *J. Am. Chem. Soc.* **113**, 9455–9458.

